# Management of Unintended Pregnancy, Complicated by the Presence of Intrauterine Contraceptive Device and a Large Uterine Fibroid: A Case Report

**DOI:** 10.7759/cureus.79466

**Published:** 2025-02-22

**Authors:** Dimitris Marin Argyriou, Emmanouil M Xydias, Elias Tsakos, Ioannis Thanasas, Apostolos C Ziogas, Cristian Furau

**Affiliations:** 1 Obstetrics and Gynaecology, "Vasile Goldiș" Western University of Arad, Arad, ROU; 2 Obstetrics and Gynaecology, EmbryoClinic IVF, Thessaloniki, GRC; 3 Obstetrics and Gynecology, General Hospital of Trikala, Trikala, GRC; 4 Obstetrics and Gynecology, University of Thessaly, Larissa, GRC; 5 Obstetrics and Gynaecology, Arad County Clinical Hospital, Arad, ROU

**Keywords:** hysterectomy, intra-uterine contraceptive device, leiomyoma, termination of pregnancy, unintended pregnancy

## Abstract

Unintended pregnancy following placement of an intrauterine device is a rare occurrence; however, it entails increased obstetrical risks if the device remains within the uterine cavity. This already challenging condition may be exacerbated by additional gynecological conditions, which would be of little or moderate risk on their own. In this report, we present such an exceedingly rare case, namely that of a 42-year-old woman who presented with unintended pregnancy, the presence of an IUD in the endometrial cavity, and a large (>10 cm) uterine fibroid. This report highlights the importance of proper patient counseling on the potential failure of IUDs and the need for frequent follow-up assessment of its placement, along with general gynecological assessment, in order to avoid the occurrence of complex gynecological pathology.

## Introduction

Unintended pregnancy, meaning unwanted or mistimed pregnancy at the time of conception, is a frequent occurrence in the modern world [1]. It is estimated that up to 46% of the approximately 200 million pregnancies occurring annually all over the world may be unintended [1]. However, data suggest that the global trend of unintended pregnancy is decreasing as time passes [2], thanks in large part to the effectiveness, variety, and availability of contraception methods [3].

Amongst the various types of contraception options, intrauterine devices (IUDs) are a particular favorite of numerous women worldwide. Constituting a safe, non-surgical, long-term (up to 10 years for copper and 5 years for levonorgestrel IUD) and reversible option, copper or levonorgestrel-based IUDs are an ideal mode of contraception for a wide range of women, from adolescent nulliparas to older multiparas [4]. Their placement is simple and safe, and they are associated with very few and rare adverse effects and complications, with the most common being displacement, which is preventable with proper training of healthcare providers and usually occurs within three months of placement, thus allowing for early detection and correction [5]. Furthermore, IUD failure rates are low, at an estimated 0.8% for copper IUDs and 0.2% for levonorgestrel-releasing IUDs, making them cumulatively up to 99% effective in preventing pregnancy [6].

However, despite their availability and relative reliability, IUDs can still fail, resulting in unintended pregnancy, the management of which may be complicated by the presence of the IUD itself as a foreign body within the endometrial cavity, with many potential adverse outcomes [7]. Effective management of such cases may be further complicated by other gynecological pathologies, such as uterine fibroids, which are quite common and thus have a small chance of occurring concomitantly with IUD failure and pregnancy [8]. Uterine fibroids, being common benign tumors of the uterus, frequently affect its anatomy and functionality and are, therefore, likely to affect pregnancy progression and complicate any potential intervention in the area. Even rare would be the occurrence of large fibroids, in particular, exceeding 10 cm, which may by themselves already affect the outcome of gestation [9], and thus, their impact would be maximized in concurrence with the presence of intrauterine IUD.

The management of such complex cases would constitute a challenging endeavor even for experienced Gynaecologists and would be highly dependent upon the individual circumstances of the patient, center of care, and Medical team. In this report, we present such a rare case of unintended pregnancy occurring concomitantly with a placed IUD and a large uterine fibroid, along with its management.

## Case presentation

**Figure 1:**
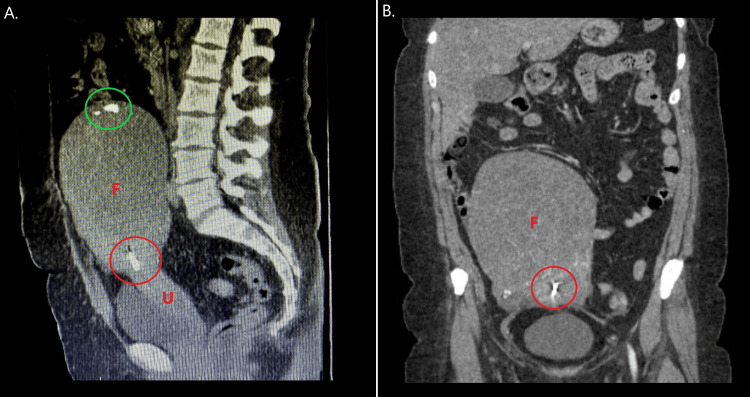
Multi-planar Computed Tomography of the patient one year prior to her presentation. A: Saggital plane, B: Coronal plane F: Fibroid, U: Uterus, red circle: intra-uterine copper device causing visual artifacts, green circle: calcifications

A 42-year-old woman was admitted to the Department of Obstetrics and Gynaecology of the Emergency Clinical Country Hospital of Arad in order to follow up on the discovery of an unintended pregnancy. The patient was gravida 2 and para 1, within the 9th gestational week on admission, class I obese (BMI: 31 kg/m^2^), and had no notable clinical symptoms. Her obstetrical history included her previous pregnancy 10 years prior, which had resulted in the cesarean delivery of a healthy neonate. Two years following her delivery (8 years prior), desiring long-term contraception, she underwent successful multiload type copper IUD placement. Her gynecological history included the incidental discovery of a large pelvic mass during a routine gynecological assessment a year prior to her current admission. Additional assessment by CT scan confirmed the presence of a large, 12x17.5 cm uterine fibroid with endometrial cavity distortion and calcifications (Figure 1); however, given the absence of clinical symptoms, she was discharged and advised to pursue regular monitoring. Her medical history included known chronic hypertension, under treatment with rilmenidine and light smoking (on average 6 cigarettes per day).

**Figure 2:**
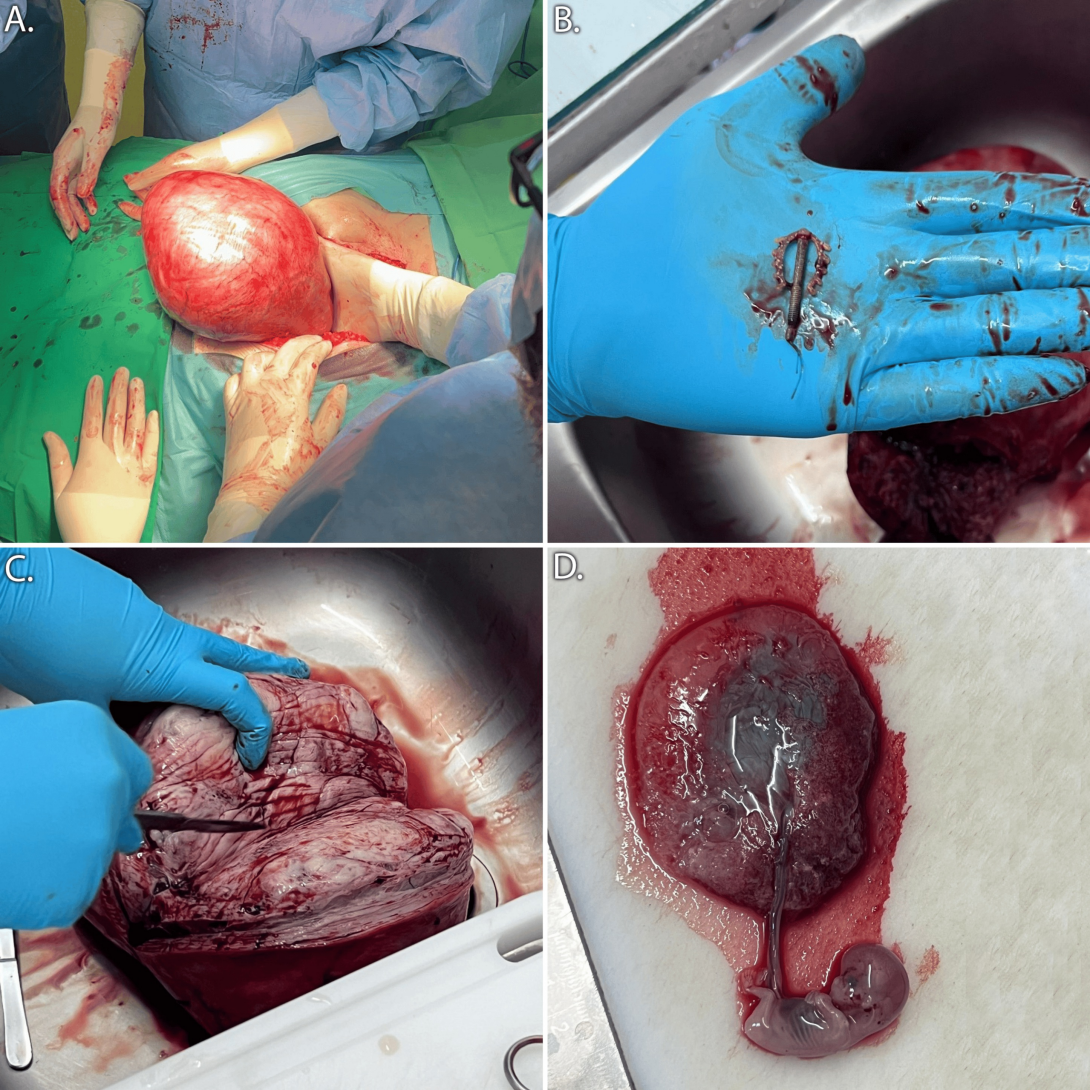
Surgical images during and after subtotal hysterectomy. A: Intra-operate manipulation of the enlarged uterus, B: Copper intrauterine device retrieved from the endometrial cavity, C: Bisected uterine fibroid, D: Retrieved placenta and fetus.

Physical examination on her admission showed a highly enlarged uterus with an upper palpable margin near the right subcoastal area. Transvaginal ultrasound confirmed the presence of an intrauterine gestational sac, along with a displaced IUD, a clear pouch of Douglas, and the known fibroid. After thorough consultation regarding the elevated risk of continued pregnancy due to the presence of the large fibroid and IUD within the endometrial cavity, in addition to the patient's own preference, a decision was made to terminate the pregnancy and perform surgery. Under general anesthesia, the patient underwent an abdominal subtotal hysterectomy. Sub-umbilical, midline incision was performed, and the significantly enlarged uterus was identified and dissected. The uterus and fibroid were removed, the former at the level of the isthmus, preserving the uterine cervix in accordance with the patient's wishes. The remaining stump was securely sutured in two layers, and so was the abdominal wall. The skin was sutured using the endodermic technique. From the retrieved surgical specimens, the IUD was located and removed; the embryo and placenta were separated and extracted from the uterus and sent along with the remaining specimens for histopathologic analysis (Figure 2). The total operative time was 120 minutes, and the estimated blood loss was 300 ml. The patient had no early postoperative complications, made an uneventful recovery, and was discharged three days later.

Histopathologic assessment confirmed the presence of a large intramural uterine fibroid, 14 cm in diameter, with typical characteristics, including intertwining smooth muscle fibers separated by well-vascularized connective tissue and a well-defined border. Mitoses were scarce, and no areas of atypia or hyaline dystrophy were present. Examination of the uterus revealed typical findings of partially decidualized endometrium, with focal hemorrhagic necrosis and associated partial infiltration by granulocytes, and the myometrium had multiple small uterine fibroids. Examination of the fetus and placenta indicated male phenotype and edematous, partially avascular chorionic villi.

## Discussion

The present case report demonstrates a rare instance of the occurrence of complicated pathology, along with pregnancy and the inherent management challenges that come with such cases. In general, IUD is a reliable contraception method employed by numerous women worldwide due to its long-term effectiveness, low cost, and low failure rate [5]. Despite that, the possibility of pregnancy cannot be entirely eliminated, and should it occur, it comes with an independently increased risk of adverse effects, regardless of whether or not the IUD is removed. In particular, pregnant women with retained copper IUD have a 6 times higher risk of spontaneous abortion, over 2.5 times higher risk of preterm birth, and 6 times higher risk of chorioamnionitis compared to normal pregnancy [7]. In the case of levonorgestrel retained IUD, there is also an increased risk of ectopic pregnancy occurrence, affecting over 60% of women with pregnancy concurrent with IUD [10].

Even if a woman wishes to continue her pregnancy, contrary to the present case, removal of the IUD is advised, which can be quite challenging and may require referral to ultrasound assessment in case of displacement and absence of strings [11]. However, even if removal is achieved, the risk of the aforementioned adverse effects may be reduced, but the risk does not reach the baseline levels associated with normal pregnancy. In particular, the risk of preterm birth is reduced from 2.5 times higher in retained IUD to 2 times higher in removed IUD compared to normal pregnancy, and the risk of chorioamnionitis from 6 to 3 times higher [7], a fact that further complicates decision-making and one that was considered and discussed with the patient of the present case during consultation. Despite all that, pregnancy with retained or removed IUD may certainly lead to a viable newborn [7], and thus, it could be pursued in cases other than the present one, where ongoing pregnancy ends up being desired after all or social and religious beliefs apply.

Similar cases of pregnancy concurrent with IUD have been reported previously in the literature, with the main outcome being spontaneous abortion or medical termination of pregnancy in the end [12]. However, the present case was further complicated by the presence of a large uterine fibroid, a known risk factor for adverse pregnancy outcomes. Namely, in a large cohort study examining obstetrical outcomes in patients with uterine fibroids over 10 cm in diameter, the cesarean section rate was 56%, all types of maternal morbidity were 62.7%, and neonatal morbidity was 18.4% [9]. The increased risk of maternal morbidity was, in fact, independent of gestational age, mode of delivery, and history of previous cesarean section [9], indicating a significant independent impact of the presence of these fibroids in the normal progression and outcome of pregnancy. Further comparative studies examining large fibroids (>5cm) confirmed these findings, with the study group having over 2 times higher risk for cesarean section, with additional findings, such as 2 times higher risk for post-partum hemorrhage and nearly 6 times higher risk for uterus atony [13]. These would likely be even more pronounced in the present case, where the fibroid's diameter was 14 cm if the patient had decided to proceed with the pregnancy.

Furthermore, our patient in the present case had completed her family plan and did not intend to attempt conception in the future, which contributed to the recommendation of subtotal hysterectomy over myomectomy, which would be the intervention of choice for similar cases where fertility preservation was desired. Excluding the patient's own wishes, myomectomy, being more technically complex, is associated with a 61-94% increase in complication rate, depending on surgical complexity, compared to abdominal hysterectomy [14]. Finally, hysterectomy has been associated with improved patient quality of life and reduced symptom severity following surgery, compared with myomectomy, therefore, it should be the method of choice in cases such as the present one.

The present case report describes the rare occurrence of two complex pathologies concurrently with pregnancy. The management of this case was made easier by the patient not desiring to continue the pregnancy and not wishing to preserve her fertility, however, it could be significantly more challenging if this was not so. Regardless, IUD remains a generally reliable, long-term method of reversible contraception and is generally associated with a low risk of adverse effects [5]. However, patients should be sufficiently counseled to attend follow-up visits after IUD insertion early at six weeks, with additional follow-up visits after the 5-year point to exclude malposition or displacement and replace expired IUDs [15], thus ensuring continued effective contraception. The presence of large fibroids, while not a contraindication to pregnancy, is certainly associated with more adverse effects and should, therefore, be addressed if possible prior to conception, with both open and minimally invasive approaches (particularly robotic approach) being viable options [16]. Even if pregnancy occurs unexpectedly, prior to myoma treatment, close obstetrical monitoring and combined cesarean delivery with myomectomy can be an effective and safe option [17].

## Conclusions

This report presented a rare case where three major gynecological pathologies occurred concomitantly, complicating management and mandating decisive action. Management could have been even more challenging if continued pregnancy and fertility preservation were desired by the patient, introducing additional challenges. While this report is very rare, and the management strategy is highly individualized and not easily generalizable, there are a few conclusions with potential for wider application. Firstly, this report highlights the need for proper patient education regarding the failure rate of IUDs and the need for follow-up after its placement, as many women usually neglect to change it in time, thus resulting in inadequate contraception and an increase in unintended pregnancy risk. Secondly, this report exemplifies the value of frequent gynecological examination for common conditions such as uterine fibroids, as they may remain asymptomatic for many years while steadily growing, resulting in an increased risk of concurrence with additional gynecological pathologies. Finally, this report demonstrates the need for thorough patient counseling and consultation regarding available treatment options in case of complex pathology and the need for treatment strategies to be as compliant as possible with the patient's own family plan and desire for future fertility.
